# A systematic review of efforts to predict day of surgery cancellation

**DOI:** 10.1097/EJA.0000000000002370

**Published:** 2026-02-25

**Authors:** Thomas Sardesai, Laura Hobbs, Caroline Phillips, Gauri Ang, Tom Bashford, Katharina Kohler, Daniel Stubbs

**Affiliations:** From the University of Cambridge School of Clinical Medicine (TS), The International Health Systems Group, University of Cambridge Department of Engineering (LH, TB, KK), East and North Hertfordshire NHS Trust (LH), The Perioperative, Acute, Critical, and Emergency Care Section (PACE), University of Cambridge Department of Medicine (CP, GA, DS), Cambridge University Hospitals NHS Foundation Trust (TB, KK, DS)

## Abstract

**BACKGROUND:**

Day of surgery cancellation (DOSC) for elective surgery occurs in 18% of elective surgeries worldwide with resultant impacts on patients and healthcare systems. Accurate prediction of such cancellations could yield significant benefit.

**OBJECTIVE:**

This systematic review synthesises evidence to inform future efforts at gold-standard statistical modelling.

**DESIGN:**

Systematic review and qualitative synthesis using the ‘Synthesis Without Meta-Analysis’ (SWiM) framework.

**DATA SOURCES:**

MEDLINE, Embase, Scopus and Web of Science, 2013–2024

**ELIGIBILITY CRITERIA:**

Studies were considered eligible if they presented the development, validation or update of a model to predict DOSC. Risk of bias for included studies was assessed using the prediction model risk of bias assessment tool (PROBAST). Data was collected on included variables, method of prediction, whether prediction was made at the level of the patient or the system, and training and assessment processes.

**RESULTS:**

Literature searching identified 7154 unique studies, 6 of which were included in the final synthesis. These studies encompassed total of 759 337 elective surgical procedures, of which 47 609 were cancellations, across a variety of adult and paediatric surgery. Methods of prediction included logistic regression models, machine learning, and spatial regression models. These used demographic, socioeconomic, comorbidity, surgical, appointment and other factors as predictors. The best discrimination achieved by models in each study, quantified by area under the receiver operator curve, ranged from 0.75 to 0.92 in development and 0.70 to 0.74 in validation datasets. One study demonstrated better calibration at the census tract level of spatial regression models when incorporating local survey data compared with individual-prediction-aggregation models.

**CONCLUSIONS:**

Models to predict DOSC identified in this review demonstrated moderate discrimination ability but there was significant heterogeneity between studies and reports of calibration were limited. This review serves as a valuable synthesis of current models to predict DOSC and should serve as a core reference for emerging studies in this field.

**KEY POINTS**

- This systematic review identified models from six studies to predict day of surgery cancellation, but these demonstrated only moderate ability to discriminate patients.
- Only one identified model reported measures of both discrimination and calibration.
- Predictive models identified used logistic regression, machine learning and spatial regression methods.
- Predictive variables used in models included demographic, socioeconomic, comorbidity, surgical, appointment and other factors.
- Further model development in a European context, reporting both discrimination and calibration would be valuable before models are implemented into clinical settings.

## Introduction

### Rationale

Day of surgery cancellation (DOSC) for elective surgery is a challenge for both patients and healthcare providers.^1^ Cancellations directly affect patients through prolonging physical disease and anxiety, as well as introducing problems with social factors such as unnecessary absence from work, childcare and transport. Health services suffer wasted resources, prolonged waiting lists, and poorer clinical outcomes.^2,3^

Numerous variables contribute to DOSC, including patient factors (e.g. changes in medical condition, uncontrolled comorbidities) and health service pressures (e.g. staff or bed shortages), necessitating a systems approach to understand and address the issue.^2,3^ A 2020 systematic review suggested DOSC occurs in 18% of elective surgeries worldwide, predominantly due to lack of available operating theatres, staffing and changes in patient condition.^4^

Predictive models can be used to improve patient care.^5^ Theoretically, DOSC may be predicted by statistical models combining patient and system factors to estimate the probability of cancellation at the level of the patient (whether a particular patient experiences DOSC) or the system (the number of patients who may require cancellation on a given day). The chosen output in each case would depend on the modelling approach, selected variables, data availability, and study aim. Well performing models of either format could create opportunities for intervention. Patients at a ‘high risk’ of cancellation could be counselled in advance, or given the opportunity to rearrange their admission, whereas days with high numbers of cancellations forecast could trigger hospitals to open contingency areas or re-prioritise cases. To realise such ambitions, it is vital that well performing models are identified (to permit their validation in external settings), and that current knowledge is synthesised to inform future statistical development.

### Objectives

When generating a prediction model, best practice stresses the importance of taking a structured approach to identifying relevant explanatory variables before commencing model development.^6^ Recognising the potential patient and system benefits of accurate DOSC prediction, this systematic review evaluates existing examples of such model development^7^ to synthesise the current state of the art in the prediction of DOSC. It assesses the methods used, the breadth of investigated explanatory variables and the performance of models. It serves as a resource for future prediction model development and validation studies. Accurate prediction of DOSC and its integration into routine practice could yield significant benefit to both patient experience and hospital flow.

## Methods and analysis

**Fig. 1:**
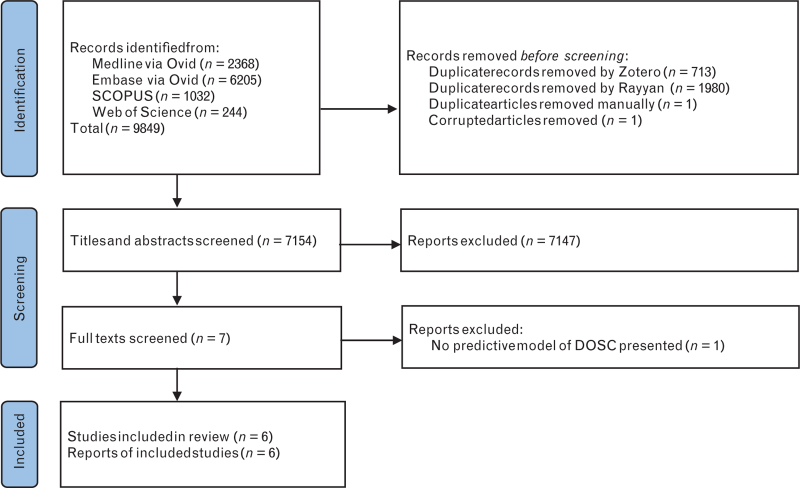
PRISMA flow chart. PRISMA, Preferred Reporting Items for Systematic reviews and Meta-Analyses.

This systematic review was conducted and reported following the PRISMA (Preferred Reporting Items for Systematic Review and Meta-Analysis) guidelines (Fig. 1).^8^ This review was registered with PROSPERO (CRD42023478984), following a published protocol.^9^ No ethical approval was required.

### Eligibility criteria

We included peer-reviewed journal articles detailing the development, validation, updating, and implementation of models to predict DOSC in which the methods, variables and performance are reported. Inclusion and exclusion criteria are detailed in the protocol for this review^9^ and summarised in Table 1. These were validated in pilot screening of 200 randomly selected studies by TS and confirmed by decision with two other authors (K.K., D.J.S.). We only included studies where a formal statistical model to predict DOSC was formulated as defined by the prediction model risk of bias assessment tool (PROBAST).^7^ We interpret a formal statistical model to comply with the development or validation stages of this document or the derivation and assessment of a model to address a specific research aim.^10^ We did not include ‘predictor-finding’ or simple associative studies that did not proceed to formal statistical synthesis.

**Table 1:** Inclusion and exclusion criteria

	Criteria	Includes	Excludes
0	English language	English language	
1	Publication type	Peer-reviewed journal articles	Magazine articles Conference abstracts Theses
2	Study type	Development, validation and updates of models Novel models and implementation studies If the same model is published multiple times, include the first paper and an implementation study if available	Review articles Systematic reviews Meta-analyses Discussion papers Subsequent publications of models without implementation
3	Subject	Day of surgery cancellation	
4	Method	Risk prediction, statistical model	Predictor-finding studies
5	Outcome 1	Method of prediction specified	
6	Outcome 2	Reports the variables used in the model	Variables kept secret
7	Outcome 3	Measures the performance of the model	

### Information sources and search strategy

The search strategy included papers from electronic databases: Medline (via Ovid), Embase (via Ovid), Scopus and Web of Science. Databases were searched in the timeframe from 1 January 2013 to 13 November 2024, limited to English language. The full search strategy is available in the published protocol for this review.^9^

### Selection process

Studies were uploaded to Zotero version 6.0.27^11^ and duplicates removed using inbuilt features. Titles and abstracts were screened by two independent authors using the online platform Rayyan^12^ and conflicts arbitrated by a third senior author (D.J.S. or K.K.). Full text screening was carried out by two authors independently using Rayyan, recording reasons for exclusions, and conflicts were resolved by D.J.S. or K.K. A PRISMA flowchart was generated (Fig. 1).

### Data collection process, data items and effect measures

Data extraction was carried out by two independent authors. Where the data parameters of interest were stated to have been collected but not reported in the published paper or supplementary material, Supplemental Digital Content, study investigators were sent a request by E-Mail. Data were extracted pertaining to the method and level of prediction, cohort characteristics, setting, method of data collection for model training, variables included, model assessment and validation method and performance. The full list of items for extraction are detailed in the protocol for this review^9^ and the characteristics of each study reported in Table 2.

**Table 2:** Included study characteristics

Study	Type of model	Method(s) of prediction	Number of patients	Level of prediction	Setting + dates	Data source	Type of surgery	Other demographics
Wongtangman *et al.*, 2022^16^	Development and validation	Backwards stepwise logistic regression Youden's J-statistic for dichotomisation cutoff	246 612 development (21 243 cancellations) 58 662 validation (5115 cancellations)	Individual	Montefiore Health System, New York, USA Development: Jan 2016 – Jun 2021 (excludes Mar-Jun 2020 due to Covid19) Validation: July 2021 – June 2022	Retrospective hospital records	Elective adult and paediatric	Age – not cancelled mean 51, cancelled mean 53 Sex – not cancelled 42.9% male, cancelled 49.7% male Language – not cancelled 73.9% English, cancelled 74.4% English
Turcotte *et al.*, 2024^17^	Development	Backwards conditional multivariate logistic regression	55 259 total (60% development, 40% internal validation cohort), 475 cancellations	Individual	Luminis Health Anne Arundel Medical Center, Annapolis, Maryland, USA May 2018 – March 2022	Retrospective hospital records	Elective adult	Did not include patients admitted for medical management prior to surgery 60.4% female 24.0% nonwhite
Zhang *et al.*, 2021^18^	Development	Machine learning supervised classification models (random forest, logistic regression, extreme gradient boosting-tree, support vector machine-linear, neural networks)	5125 cases, 810 cancellations	Individual	West China Hospital January 2013 – December 2014	Retrospective hospital records	Elective urology	All surgeries scheduled 1 day in advance Utilised over-sampling and under-sampling methods to improve performance
Liu *et al.*, 2019^19^	Development and validation	Machine learning classification models (naïve Bayes, multivariate logistic regression with L1 and L2 normalisation, support vector machines with polynomial and radial basis function kernels, CART and C5.0 decision trees, random forests, gradient boosted logistic regression, artificial neural networks, TensorFlow deep learning algorithm)	Burnet campus: 58 301 (training), 24 960 (test), 3088 cancellations Liberty campus: 29 729 (training), 12 703 (test), 1940 cancellations	Individual	Burnet campus and Liberty campuses of Cincinnati Children's Hospital Medical Centre, Cincinnati, USA 2012–2017	Retrospective hospital records	Elective paediatric	Rescheduled surgeries not included
Liu *et al.*, 2021^20^	Development	Nonspatial regression models (GLM, L2-normalised GLM, support vector machine with polynomial kernels, decision tree) Spatial regression models (SAR model, spatial Durbin model, SEM, spatial Durbin error model, spatial moving average, SAR confused models)	254 546 patients (229 600 geocoded to the city-block level, 17 309 geocoded to the street level) CCHMC: 88 013 surgeries, 3702 cancellations TCH: 166 533 surgeries, 10 236 cancellations	Individual prediction and census tract level prediction	5 campuses over 2 trusts: Cincinnati Children's Hospital Medical Centre and Texas Children's Hospital, USA May 2011 – May 2016	Retrospective hospital records and the American Community Survey	Elective paediatric	Rescheduled surgeries not included Tertiary and quaternary care referrals from elsewhere accepted
Li *et al.*, 2024^21^	Development	Binary logistic regression (LR) with L2 regularisation, linear support vector machines (SVM), random forests (RF), gradient boosting trees (GBDTs), extreme gradient boosting (XGBoost)	13 440 surgeries (70% development, 30% internal validation cohort), 1000 cancellations	Individual	National Center for Children's Medical Research in China, Children's Hospital, Zhejiang University School of Medicine, Hangzhou, China 2018-2021	Retrospective hospital records China Meteorological Data Sharing Service System	Elective paediatric	8632 male, 4808 female. Mean age 3.41. 63.5% from cities.

CART, Classification and Regression Trees; CCHMC, Cincinnati Children's Hospital Medical Centre; GLM, generalised linear regression model; SAR, regressive spatial autoregressive model; TCH, Texas Children's Hospital.

In training, internal and external validation datasets, measures of discrimination and calibration were measured and tabulated in Table 3. For discrimination, the primary effects measure was area under the receiver-operator curve (ROC-AUC). For calibration, effect measures included Brier score and root mean square error (RMSE).

**Table 3:** Best model discrimination and calibration in training and validation datasets

Study	Assessment in training dataset		Validation		
		
	Discrimination	Calibration	Method	Discrimination	Calibration
Wongtangman *et al.*, 2022^16^	ROC-AUC 0.79 (95% CI, 0.79 to 0. 80)	Brier score 0.067 (reliability <0.001)	External (same campus, different timepoint)	ROC-AUC 0.73 (95% CI, 0.72 to 0.73)	Brier score 0.074 (reliability *P* <0.001)
Turcotte *et al.*, 2024^17^	No information	No information	Internal (40% holdout validation cohort)	ROC-AUC 0.70 (95% CI, 0.65–0.74)	No information
Zhang *et al.*, 2021^18^	No information	No information	Internal (five-fold cross validation)	Random forest model with original sampling and backwards selection strategy – ROC-AUC 0.72 (no CI reported)	No information
Liu *et al.*, 2019^19^	No information	No information	Internal (tenfold cross-validation), internal (30% holdout validation cohort) and external (different campus)	Internal (tenfold cross validation) – Burnet GBL ROC-AUC 0.78 (95% CI, 0.76 to 0.80); Liberty GBL ROC-AUC 0.74 (95% CI, 0.76 to 0.77). Internal (30% holdhout) Burnet GBL ROC-AUC 0.79 (no CI reported) Liberty GBL ROC-AUC 0.75 (no CI reported). External - LR+L1 model outperformed GBL. Liberty model validated on Burnet data ROC-AUC 0.73 vs. 0.72 for GBL (no CIs reported), Burnet model validated on Liberty data ROC-AUC (0.74 vs. 0.73 for GBL (no CIs reported)	No information
Liu *et al.*, 2021^20^	Not applicable	No information	Internal (tenfold cross validation)	Not applicable	CCHMC – L2-normalised GLM RMSE 1.3 (95% CI, 1.21 to1.39)% Geospatial models outperformed the individual-prediction-aggregation model (RMSE 4.19%, *P* < .001, 95% CI, 4.18 to 4.20) TCH – L2-normalised GLM RMSE 1.31% (95% CI, 1.26- to.35) Geospatial models outperformed individual-prediction-aggregation models.
Li *et al.*, 2024^21^	No information	No information	Internal (tenfold cross validation)	XGBoost model – ROC-AUC 0.92 (no CI reported), sensitivity 0.84, precision 0.84, F1 score 0.84; and accuracy 0.84	No information

CCHMC, Cincinnati Children's Hospital Medical Centre; DOSC, day of surgery cancellation; F1 score, a metric of positive predictive value and sensitivity; GBL, gradient-boosted logistic regression; GLM, generalised linear regression model; RMSE, root mean square error; ROC-AUC, area under the receiver operator curve; TCH, Texas Children's Hospital; XGBoost, extreme gradient boosting.

### Study risk of bias assessment

Risk of bias for each study was assessed using the PROBAST tool^7^ by two independent authors, with conflicts resolved by D.S. (Table 4). Missing results for parameters of interest resulted in the study being excluded from the analysis of that parameter Supplemental Table 2.

**Table 4:** PROBAST risk of bias assessment summary table

Study	Risk of bias				Applicability			Overall	
			
	Participants	Predictors	Outcome	Analysis	Participants	Predictors	Outcome	Risk of bias	Applicability
Wongtangman *et al.*, 2022^16^	+	+	+	?	+	+	+	+	+
Turcotte *et al.*, 2024^17^	+	+	+	–	+	+	+	–	+
Zhang *et al.*, 2021^18^	+	+	+	–	+	+	+	–	+
Liu *et al.*, 2019^19^	+	+	+	+	+	+	+	+	+
Liu *et al.*, 2021^20^	+	+	+	–	+	+	+	–	+
Li *et al.*, 2024^21^	+	+	?	+	+	+	?	?	?

“+” indicates low risk of bias, low concern regarding applicability; “?” indicates unclear risk of bias, unclear concern regarding applicability; “–“ indicates high risk of bias, high concern regarding applicability.

### Synthesis methods

Due to the small number of studies and heterogeneity, data were not subject to meta-analysis but were instead guided by SWiM (synthesis without meta-analysis) guidelines.^13^ Results were prioritised based on results of the PROBAST risk of bias assessment,^7^ then use of external validation, then breadth of surgical types. Where results were not reported or reported incompletely (e.g. ‘results nonsignificant’ rather than giving a figure), this was acknowledged as a source of reporting-bias^14^ and studies were excluded from the analysis of the parameter.

Per the protocol,^9^ studies were to be sub-grouped for analysis by setting and prediction level (patient-level vs. system-level). Analysis would be carried out on all studies in aggregate, two sub-groups by prediction level, two sub-groups by setting and four sub-groups by prediction level and setting. Since no included studies predicted at the system level, analysis was performed in aggregate and in sub-groups by setting. Setting was defined by the World Bank economic categories by gross national income: low, low-middle, upper-middle and high income countries. Our protocol aimed to stratify by LMIC (low, low-middle and upper-middle income countries) vs. HIC (high-income countries).^15^ However, since no studies from low or low-middle income countries were identified, studies were stratified into those from China, an upper-middle income country (UMIC)^15^ and those from the United States, a HIC.^15^ Sensitivity analysis excluded internal-only validation.

## Results

### Study selection

Literature searching identified a total of 9849 records which were uploaded to Zotero. 2695 duplicated records were removed, and 7154 unique studies were screened in duplicate by T.S., C.P. and L.H. using Rayyan software^12^ according to the selection criteria.^9^ After title and abstract screening, seven studies were carried forward to full text screening by T.S. and C.P. One article was excluded for not presenting a predictive model of DOSC, leaving six for assessment.^16–21^ The PRISMA flowchart in Fig. 1 summarises this process.

### Risk of bias in studies

Six included articles were assessed for risk of bias and applicability using the PROBAST tool,^7^ the results of which are summarised in Table 4.

All studies were conducted using retrospective hospital records. Exclusions from datasets were felt to be appropriate, including cases during COVID-19 surges with reduced surgical capacity,^16^ cases not scheduled in advance^17^ and previously rescheduled cases.^19^ Predictors were determined using standardised methods such as International Classification of Disease 10th Edition codes in patient records on the operative date.^17^ Predictors were available at the time of intended use in electronic records or partner sources (e.g. weather reports).^19,20^ Data were largely handled appropriately but continuous data was occasionally dichotomised,^16^ univariate analysis was used in selecting predictors^17,20^ and calibration was inconsistently reported.^17,18,20,21^ The number of DOSC events in training data ranged from 475^17^ to 26 358^16^ with a minimum events per variable of 23.^17^ Overfitting and optimism were accounted for using bootstrapping,^16^ holdout cohorts,^17^ internal fivefold or tenfold cross validation.^18–21^

Regarding applicability, our review question aims to identify what has been used to predict DOSC. Despite some predictors being unique to the study area such as U.S.-specific insurance information^16,19,20^ and geospatial data specific to the development dataset,^20^ the concept of using financial information or geographic area to infer socioeconomic data as predictors is applicable to diverse settings.

### Results of individual studies – levels of prediction and validation

Each study presented the development of a predictive model of DOSC at the individual level, detailed in Table 2. Liu *et al.*^19^ generated two sets of predictive models separately, one from each of the Burnet and Liberty campuses of the Cincinnati Children's Hospital Medical Centre (CCHMC). Liu *et al.*^20^ also generated census-tract-level predictions of DOSC per geographic area by aggregating information from the 2011 to 2015 American Community Survey.^22^ Notably, the models presented in Liu *et al.*^19^ and Liu *et al.*^20^ were derived from the same dataset.

### Results of individual studies – methods of performance

Discriminative performance, assessed by maximum ROC-AUC, ranged from 0.70 to 0.92 in training, internal and external validation datasets,^16–21^ details of which can be found in Table 3. Specifically, logistic regression by Wongtangman *et al.*^16^ achieved a ROC-AUC of 0.79 (95% CI, 0.79 to 0.80) in the training dataset and 0.73 (95% CI, 0.72 to 0.73) in an external validation dataset. Logistic regression by Turcotte *et al.*^17^ achieved a ROC-AUC of 0.70 (95% CI, 0.65 to 0.74) in the 40% holdout internal validation dataset. Random forest machine learning by Zhang *et al.*^18^ achieved a mean ROC-AUC of 0.69 (standard deviation 0.03) with the maximum ROC-AUC of 0.72 (no CI reported) achieved using a backwards selection strategy during internal five-fold cross-validation. Gradient-boosted logistic regression (GBL) models by Liu *et al.*^19^ achieved ROC-AUCs of 0.78 (95% CI, 0.76 to 0.80) in the Burnet dataset and 0.74 (95% CI, 0.73 to 0.77) in the Liberty dataset during internal tenfold cross-validation. The same models achieved ROC-AUCs of 0.79 (no CI reported) and 0.75 (no CI reported) respectively when tested on the 30% holdout dataset and 0.73 and 0.72 (no CI reported) respectively during external validation on the opposite campus’ complete datasets. Logistic regression with L1-normalisation of included vectors (LR+L1) marginally outperformed GBL models during external validation, achieving ROC-AUCs of 0.74 and 0.73 (no CI reported) when the Burnet-trained model and Liberty-trained model were validated on opposite datasets respectively. The extreme gradient boosting (XGBoost) model by Li *et al.*^21^ yielded a ROC-AUC of 0.92 (no CI reported) during internal tenfold cross-validation but no external validation was reported. Of the above studies, only Wongtangman *et al.*^16^ reported a measure of calibration, achieving a Brier score of 0.074 (reliability *P* < 0.001).

Liu *et al.*^20^ compared the calibration of nonspatial regression models to spatial regression models specific to the catchment areas of the five campuses of the CCHMC and Texas Children's Hospital (TCH) using root mean square error (RMSE). The models which predicted DOSC rates per geographical location at the census tract level were compared to models predicting at an individual level aggregated to the census tract level. The lowest RMSE was achieved by the L2-normalised generalised linear regression model (GLM) at 1.3 (95% CI, 1.21 to 1.39)%, a nonspatial regression model for the CCHMC data set. All models outperformed the individual-prediction-aggregation model (RMSE 4.2, 95% CI, 4.18 to 4.20)%, suggesting incorporation of geographic data improved model calibration. For the TCH data set, the lowest RMSE was also achieved by the L2-normalised GLM at 1.31 (95% CI, 1.26–1.35)%.

### Results of individual studies – variables included in predictive models

**Fig. 2:**
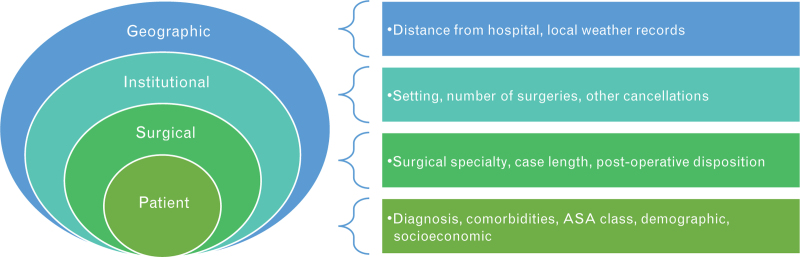
Predictive variables in included models. Further information in Supplementary Table 1.

**Fig. 3:**
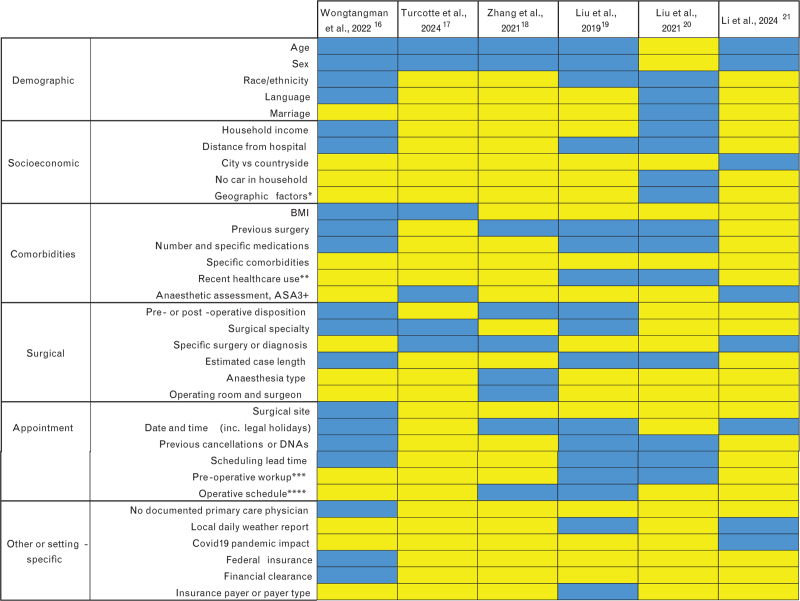
Heatmap of predictive variables used in predictive models of DOSC.

Variables used for prediction could be divided into demographic, socioeconomic, comorbidities, surgical, appointment and other factors, detailed in Figs. 2 and 3. Demographic factors were used in all six studies, including age, sex,^16–19,21^ race, ethnicity,^16,19,20^ language spoken,^16,20^ and marital status.^20^

Blue, predictor used; yellow, predictor not used; ASA, American Society of Anaesthesiology; DNA, did not attend. * Total population in the area, vacant houses, median home value, population with low educational attainment, rented houses, household overcrowding. ** Emergency room attendance, hospitalisations within 6 months. *** Office visits, phone calls, history and examination. **** WSHSB, whether the surgeon has surgery before the case; NSOD, number of surgeries in the OR on the day; ONS, order number of surgery in the list; WMSD, whether it is the main surgery day (a feature of the organisation of the operating schedule in the study by Zhang *et al.* in which each surgeon has a main operating day to which their cases are assigned); WC, whether other surgeries have been cancelled during that operating list; work-in case, whether other surgeries have been cancelled during that operating list.

Socioeconomic factors were included in four studies, including factors related to household income, address, and distance to the hospital.^16,19–21^ Although some health insurance predictors used were U.S.-specific such as federal insurance,^16,20^ insurance-based systems are used across multiple European and wider contexts and these predictors could be substituted if no equivalent is available in a new setting. Liu *et al.*^20^ also included location-specific socioeconomic factors including median home value, median household income and proportion of houses rented in the area. Li *et al.*^21^ also distinguished between patients living in cities vs. the countryside.

Comorbidities were used in all six studies, including body mass index (BMI),^16,17^ previous surgery,^16,18–20^ specific comorbidities,^16,17,21^ and preanaesthetic assessment such as high American Society of Anaesthesiology score (ASA3+).^17,21^

Surgical factors were used in all six studies. These included the type of surgery,^16–19^ underlying diagnosis,^21^ estimated duration of surgery,^16,19,20^ and pre or postoperative disposition.^16,18,19^ Appointment-related factors were included in five studies, including factors relating to previous cancellations or nonattendances,^16,18–20^ pre-operative information such as lead time and phone call attempts,^16,18–20^ the date and time of surgery,^16,18,19,21^ and scheduling information such as use of a free-standing surgical centre, number of surgeries scheduled, and the order number.^16,18,19^ Other factors were used by three studies, including the local circulating load of respiratory, gastrointestinal and febrile pathogens,^19^ detailed daily weather records,^19,21^ and lack of a documented primary care physician.^16^

### Subgroup analysis – analysis by income setting

In the two studies from UMICs,^18,21^ there were 18 565 cases of which 1810 were cancellations to generate predictive models, the best of which had ROC-AUCs ranging from 0.72 to 0.92 during internal validation but no external validation was reported. In the 4 studies from HICs,^16,17,19,21^ there were more datapoints used to train models, comprising 740 772 cases of which 45 799 were cancellations. The best HIC-developed models’ ROC-AUCs ranged from 0.70 to 0.79 during internal validation and 0.73 to 0.74 during external validation.

Logistic regression models with different feature selection and weighting strategies were predominantly reported in HIC studies^16,17,19^ and decision-tree machine learning models in UMIC studies.^18,21^ The predictive variables were broadly similar, however there were variables exclusive to HICs, including BMI,^16,17^ race,^16,19,21^ financial and insurance information^16,19,20^ and pre- and postop disposition^16,19^ whilst the only study including type of anaesthesia was from a UMIC.^18^

### Sensitivity analysis – excluding internal-only validation

Two studies comprising 184 355 cases of which 10 143 were cancellations reported external validation.^16,19^ Wongtangman *et al.*^16^ utilised a logistic regression model in a validation cohort collected at a different time to the original sample, achieving a maximum ROC-AUC of 0.73 (95% CI, 0.72 to 0.73) and calibration Brier score of 0.074 (reliability < 0.001). Liu *et al.*^19^ used LR+L1 and GBL models in validation of models on the opposite dataset from which they were developed, achieving a maximum AUC of 0.74 when the Burnet-trained LR+L1 model was validated on Liberty data (no CIs reported). The models and performance of models for which external validation were reported were similar to the models for which only internal validation was reported.

### Heterogeneity analysis

Included studies used different populations and surgical specialties to develop models. Whilst Wongtangman *et al.*^16^ included adult and paediatric cases, Turcotte *et al.*^17^ included only adult cases, Zhang *et al.*^18^ included only urology cases and Liu *et al.*,^19^ Liu *et al.*^20^ and Li *et al.*^21^ included only paediatric cases. The models identified in this review were derived from the USA and China, countries with significant differences in their healthcare systems. These include private vs. public ownership of healthcare facilities and employer or individually funded health insurance vs. predominantly government-funded insurance. This was reflected in the use of financial and insurance information as predictors of DOSC in multiple models from the United States but not from China. Predictive variables differed between studies but could broadly be categorised as detailed in Figs. 2 and 3. Each model included demographic, comorbidity and surgical factors but in the studies by Turcotte *et al.*^17^ and Zhang *et al.*,^18^ the two models were developed without paediatric cases, and did not include socioeconomic factors. Furthermore, Turcotte *et al.*^17^ did not include appointment-related factors, perhaps due to differences in populations.

## Discussion

We present a detailed synthesis of the current literature on predicting DOSC for elective surgery. Despite the importance of DOSC to patients and healthcare providers, we only identified six studies matching our inclusion and exclusion criteria. Barring one study attempting to predict cancellations by geographical capture area, all studies focused on predicting individuals at risk of DOSC rather than estimating the daily number of cancellations for a specific surgical service. Incorporation of geographic data demonstrated potential for improved predictive performance. Models exhibited moderate discriminative performance^23^ and different measures of calibration were reported in two studies.^16,20^

Similar rates of elective surgical cancellation have been recorded in hospitals worldwide, including 13.1% in Qatar,^24^ 14.0% in Canada^25^ and 12.8% in Leicester, UK (of which 33.3% were DOSC),^26^ indicating the potential for widespread benefit from well performing models to predict surgical cancellation. Our findings provide a comprehensive source of examined predictor variables and suggest avenues for future research, such as taking a system level predictive approach. Performance of models predicting DOSC in this review demonstrated similar predictors and outcomes to the random forest model predicting surgical cancellation at any time developed by Lou *et al.*^27^ which achieved a maximum ROC-AUC of 0.68.

Predictive models have been introduced into anaesthetic practice, including a multivariable logistic regression model to predict perioperative mortality developed from the National Emergency Laparotomy Audit (NELA).^28^ If a well discriminating and calibrated model to predict DOSC was implemented during surgical scheduling, lists could be re-organised to minimise cancellations. In 2016, a single centre pilot study estimated avoidable delays from increased turnaround time between surgical cases cost £347 327 per theatre per year in lost national reimbursement tariffs and staffing costs.^29^ If suitable models to predict potential DOSC could be developed and potential theatre gaps predicted, then an opportunity to repurpose such lost time could arise. This would yield potential patient benefit (by facilitating quicker scheduling) and system benefit (by reducing waiting lists). In some cases, reserve waiting lists of patients who may be contacted in case of surgical cancellations have been implemented.^30^

With a gold-standard predictive model of DOSC, these patients could be counselled in advance about the days during which they may be contacted. Furthermore, as many as 60% of surgical cancellations may be avoidable.^31^ Where a pattern of cancellations is predicted, targeted interventions such as opening contingency areas or requesting contingency staff members could, in theory, be introduced. Implementing such an appropriately validated tool into practice is a complex system problem. The distinct barriers and facilitators to this are likely to vary by setting and is an area where complementary research into the statistical work we have synthesized here is needed.

A systematic review of 78 studies of factors associated with cancellation and delay of elective surgical procedures found hospital-related causes were predominant. These included poor scheduling, insufficient beds and operating room availability.^32^ Further hospital-related causes including inadequate staffing, equipment shortages and over-booking of operating rooms have also been identified.^25–27^ The review also identified patient and workup-related factors responsible for surgical cancellation, including being medically unfit, changes in the treatment plan and patient absence or refusal.^32^ Other patient and workup-related factors have been associated with cancellation including insufficient pre-operative investigation and abnormal laboratory results.^25^ The studies identified in our review did not report incorporation of some hospital-, patient- and workup-related causes as predictors in their models, including abnormal laboratory results or the availability of beds, staff or equipment, suggesting avenues of investigation for future model development.

A gold-standard model of DOSC would be the best available under reasonable conditions, thoroughly tested with the highest sensitivity and specificity of available methods.^33^ This would be reflected in the discrimination and calibration across surgical settings with sufficient time before predicted DOSC to make meaningful changes. Obstacles to generating such a model include the number of predictive variables, real-time availability of these variables and differences in determinants of DOSC across different settings.

Our review has a number of strengths. The number of participants in each study was large, ranging from 5125 to 305 274 across included studies.^16–21^ These studies were applicable to our review question, risk of bias was comprehensively assessed using a standardised method^7^ and the most consistently used metric for assessment was ROC-AUC, a valuable metric for evaluating the discrimination of predictive models.^34^

As with many systematic reviews we acknowledge the limitations of our search. There were only six studies identified and data were exclusively drawn from the United States and China, potentially limiting generalisability and highlighting the need for model generation in a European context. We only included studies published in English language^35^ and our literature search only included studies from the last 10 years with the intention that the returned results would be more relevant to the current organisation of healthcare. We acknowledge that some potentially relevant studies may have been excluded, however it has been established that the majority of studies on surgical cancellation have been published since 2010.^32^ The synthesis was altered postprotocol to exclude prediction level subgroup analysis due to lack of system level models reported.^9^ There was also significant heterogeneity between the studies included with regards to the populations and settings from which training data were extracted.

Our review involves a comprehensive search of four major literature databases and serves as a valuable synthesis of current models to predict DOSC and should serve as a core reference for emerging studies in this field. This review identified a lack of models predicting DOSC in European settings, and a lack of models at the system level. There was also significant heterogeneity, limiting comparison of models. There may be utility in standardising the metrics of evaluation of discrimination and calibration. Future studies could investigate predictive models of DOSC prospectively to determine when there is sufficient data availability and predictive value to put meaningful interventions in place. Furthermore, it is likely that future developments in prediction of DOSC will benefit from advancements in machine learning technology.

### Overall conclusions

Six models have been reported in the English-language literature to predict individual risk of DOSC, achieving moderate discrimination. Models utilised demographic, socioeconomic, comorbidity, surgical, appointment and other predictors including ‘nonpatient level’ geographic and weather data, highlighting that DOSC is a complex system phenomenon. This underscores the need for further research into predicting DOSC using standardised measures of discrimination and calibration.

## Supplementary Material

Supplemental Digital Content

## Supplementary Material

Supplemental Digital Content

## Supplementary Material

Supplemental Digital Content

## References

[1] Nasr A Reichardt K Fitzgerald K. Impact of emergency admissions on elective surgical workload. *Ir J Med Sci* 2004; 173:133–135. 15693381 10.1007/BF03167926

[2] Krueger CA Kozaily E Gouda Z. Canceled total joint arthroplasty: who, what, when, and why? *J Arthroplasty* 2021; 36:857–862. 33032875 10.1016/j.arth.2020.09.006

[3] Gillies MA Wijeysundera DN Harrison EM. Counting the cost of cancelled surgery: a system wide approach is needed. *Br J Anaesth* 2018; 121:691–694. 30236228 10.1016/j.bja.2018.08.002 PMC7118877

[4] Abate SM Chekole YA Minaye SY. Global prevalence and reasons for case cancellation on the intended day of surgery: a systematic review and meta-analysis. *Int J Surg Open* 2020; 26:55–63. 34568611 10.1016/j.ijso.2020.08.006 PMC7440086

[5] Tran ET Ho KM. Utility of the National Emergency Laparotomy Audit prognostic model in predicting outcomes in an Australian health system. *Anaesth Intensive Care* 2023; 51:51–58. 36475889 10.1177/0310057X221105291

[6] Leisman DE Harhay MO Lederer DJ. Development and reporting of prediction models: guidance for authors from editors of respiratory, sleep, and critical care journals. *Crit Care Med* 2020; 48:623–633. 32141923 10.1097/CCM.0000000000004246 PMC7161722

[7] Wolff RF Moons KGM Riley RD. PROBAST: a tool to assess the risk of bias and applicability of prediction model studies. *Ann Intern Med* 2019; 170:51–58. 30596875 10.7326/M18-1376

[8] Page MJ McKenzie JE Bossuyt PM. The PRISMA 2020 statement: an updated guideline for reporting systematic reviews. *BMJ* 2021; 372:n71. 33782057 10.1136/bmj.n71 PMC8005924

[9] Sardesai T Hobbs L Phillips C. Examining efforts to predict day-of-surgery cancellation (DOSC): a systematic review protocol. *J Surg Protoc Res Methodol* 2024; 2024:snae001.

[10] Steyerberg EW Vergouwe Y. Towards better clinical prediction models: seven steps for development and an ABCD for validation. *Eur Heart J* 2014; 35:1925–1931. 24898551 10.1093/eurheartj/ehu207 PMC4155437

[11] Roy Rosenzweig Center for History and New Media. Zotero. [Computer software] 2023. www.zotero.org/download [Accessed 13 November 2024].

[12] Ouzzani M Hammady H Fedorowicz Z. Rayyan-a web and mobile app for systematic reviews. *Syst Rev* 2016; 5:210. 27919275 10.1186/s13643-016-0384-4 PMC5139140

[13] Campbell M McKenzie JE Sowden A. Synthesis without meta-analysis (SWiM) in systematic reviews: reporting guideline. *BMJ* 2020; 368:l6890. 31948937 10.1136/bmj.l6890 PMC7190266

[14] Higgins JP Thomas J Chandler J. Cochrane handbook for systematic reviews of interventions version 6.4. 2023; Cochrane, www.cochrane.org/handbook [Accessed 1 November 2023]

[15] World Bank Country and Lending Groups. World Bank Data Help Desk. 2024. https://datahelpdesk.worldbank.org/knowledgebase/articles/906519-world-bank-country-and-lending-groups [Accessed 13 November 2024].

[16] Wongtangman K Azimaraghi O Freda J. Incidence and predictors of case cancellation within 24 h in patients scheduled for elective surgical procedures. *J Clin Anesth* 2022; 83:110987. 36308990 10.1016/j.jclinane.2022.110987

[17] Turcotte JJ Brennan JC Kidd G. Predictors of same day cancellation of elective surgery. *J Perioper Pract* 2024; 34:178–186. 37646416 10.1177/17504589231189349

[18] Zhang F Cui X Gong R. Key experimental factors of machine learning-based identification of surgery cancellations. *J Healthc Eng* 2021; 2021:6247652. 33688420 10.1155/2021/6247652 PMC7914093

[19] Liu L Ni Y Zhang N. Mining patient-specific and contextual data with machine learning technologies to predict cancellation of children's surgery. *Int J Med Inform* 2019; 129:234–241. 31445261 10.1016/j.ijmedinf.2019.06.007

[20] Liu L Ni Y Beck AF. Understanding pediatric surgery cancellation: geospatial analysis. *J Med Internet Res* 2021; 23:e26231. 34505837 10.2196/26231 PMC8463951

[21] Li C Li Z Huang S. Machine learning-based approach to predict last-minute cancellation of pediatric day surgeries. *Comput Inform Nurs* 2024; 42:363–368. 38453534 10.1097/CIN.0000000000001110

[22] U.S. Census Bureau. 2011–2015 American Community Survey 5-year estimates. 2016. https://www.census.gov/programs-surveys/acs/technical-documentation/table-and-geography-changes/2015/5-year.html [Accessed 13 November 2024].

[23] de Hond AAH Steyerberg EW van Calster B. Interpreting area under the receiver operating characteristic curve. *Lancet Digit Health* 2022; 4:e853–e855. 36270955 10.1016/S2589-7500(22)00188-1

[24] Sahraoui A Elarref M. Bed crisis and elective surgery late cancellations: an approach using the theory of constraints. *Qatar Med J* 2014; 2014:1–11. 25320686 10.5339/qmj.2014.1 PMC4197367

[25] Koh WX Phelan R Hopman WM. Cancellation of elective surgery: rates, reasons and effect on patient satisfaction. *Can J Surg* 2021; 64:E155–E161. 33666393 10.1503/cjs.008119 PMC8064262

[26] Sundaram K Sankaran S Amerally P. Cancellation of elective oral and maxillofacial operations. *Br J Oral Maxillofac Surg* 2007; 45:656–657. 17950963 10.1016/j.bjoms.2007.06.001

[27] Luo L Zhang F Yao Y. Machine learning for identification of surgeries with high risks of cancellation. *Health Informatics J* 2018; 26:141–155. 30518275 10.1177/1460458218813602

[28] Eugene N Oliver CM Bassett MG. Development and internal validation of a novel risk adjustment model for adult patients undergoing emergency laparotomy surgery: the National Emergency Laparotomy Audit risk model. *Br J Anaesth* 2018; 121:739–748. 30236236 10.1016/j.bja.2018.06.026

[29] Wei Ang W Sabharwal S Bhattacharya R. The cost of trauma operating theatre inefficiency. *Int J Surg* 2016; 36:S109. 10.1016/j.amsu.2016.03.001 PMC4796663 27047660

[30] Antoniou V Burke O Fernandes R. Introducing a reserve waiting list initiative for elective general surgery at a District General Hospital. *BMJ Open Qual* 2019; 8:e000745. 10.1136/bmjoq-2019-000745 PMC6711434 31523742

[31] Schofield WN Rubin GL Piza M. Cancellation of operations on the day of intended surgery at a major Australian referral hospital. *Med J Aust* 2005; 182:612–615. 15963016 10.5694/j.1326-5377.2005.tb06846.x

[32] Koushan M Wood LC Greatbanks R. Evaluating factors associated with the cancellation and delay of elective surgical procedures: a systematic review. *Int J Qual Healthcare* 2021; 33:mzab092. 10.1093/intqhc/mzab092 34100548

[33] Cardoso JR Pereira LM Iversen MD. What is gold standard and what is ground truth? *Dental Press J Orthod* 2014; 19:27–30. 10.1590/2176-9451.19.5.027-030.ebo PMC4296658 25715714

[34] Fawcett T. An introduction to ROC analysis. *Pattern Recognit Lett* 2006; 27:861–874.

[35] Moher D Pham B Lawson ML. The inclusion of reports of randomised trials published in languages other than English in systematic reviews. *Health Technol Assess* 2003; 7:1–90. 10.3310/hta7410 14670218

