# Supplementary material for: A systematic review of efforts to predict day of surgery cancellation
Source: Eur J Anaesthesiol. 2026 Feb 25;43(8):692–702. doi: 10.1097/EJA.0000000000002370 (PMC13336580; doi:10.1097/EJA.0000000000002370)
Supplement: Supplemental Digital Content [file ejanet-43-692-s002.docx]

**Supplementary table 1 – Variables included**

| **Study** | **Demographic** | **Socioeconomic** | **Comorbidities** | **Surgical** | **Appointment** | **Other** |
| --- | --- | --- | --- | --- | --- | --- |
| Wongtangman et al., 2022 | Age (over 70)  Sex (male)  Race (black)  Language (other or unknown) | Income under $75k/year  Federal insurance  Financial clearance | BMI (over 30)  Anxiety  Opioid or benzodiazepine prescription within 30 days  Previous surgery | Ambulatory  Inpatient  Specialty (general, neurosurgery, vascular, paediatric)  Estimated case length (over 1h) | Setting (free-standing ambulatory surgery centre)  Schedule (day of week, lead time over 30 days)  Previous cancellations (no-shows under 20%)  Distance from hospital  (15-30km, over 30km) | No documented primary care physician |
| Turcotte et al., 2023 | Age (under 50, 50-59)  Sex (female) | None | BMI (under 30, 30-35, 35-40)  Heart disease (MI, CHF)  Renal disease  Liver disease  ASA3+ | Surgical specialty (ortho ‘TJA, spine’, general ‘cholecystectomy, appendectomy’, gynae, thoracic, breast) | None | None |
| Zhang et al., 2021 | Age  Sex | None | Whether there has been a surgery before | Anaesthesia type  Surgery type  Operating room  Surgeon | Schedule (main surgery day, legal holiday, number of surgeries in the theatre, order number of surgery, whether surgeon has surgery before)  Number of days admitted  Whether (other) surgery is cancelled | None |
| Liu et al., 2019 | Age  Sex  Race  Ethnicity | Distance from home  Insurance payer  Insurance payer type | Number of outpatient medications  Recent ER attendance  Hospitalisations within 6 months  Previous surgery | Surgical specialty  Post-op disposition  Estimated case length | Schedule (work-in case, time, day, month, lead time)  Preoperative contact (office visits, clinic no-shows, phone call attempts, live contact reached, first and final contacts, history and exam completed)  Previous cancellations (total number, no-shows, other) | Time since original QI project  Local circulating respiratory, GI and febrile pathogens  Daily weather records |
| Liu et al., 2021 | Race (Black, African American)  Ethnicity (Hispanic, Latino)  Language (linguistic isolation)  Adults never married | Families in poverty  Population with low educational attainment  No car in household  Rented houses  Median home value  Median household income  Vacant houses  Household overcrowding  Population in the area | Number of outpatient medications before surgery  Recent ER attendance  Hospitalisations within 6 months  Previous surgeries | Estimated case length | Schedule (lead time)  Preoperative contact (office visits, clinic no-shows, number of phone call attempts)  Previous cancellations (total, no-shows, other)  Distance from hospital (driving time) | None |
| Li et al., 2024 | Age  Gender | Address  City vs Countryside | Pre-anaesthetic assessment | Diagnosis | Covid19 pandemic impact  Appointment week and month | Windspeed, rainfall, humidity, temperature and air pressure |

*BM, body mass index; MI, myocardial infarction; CHF, chronic heart failure; ASA, American Society of Anesthesiologists; ortho, orthopaedics; TJA, total joint arthroplasty; gynae, gynaecology; QI, quality improvement; GI, gastrointestinal; ER, emergency room*
